# Correlation between patient quality of life in palliative care and burden of their family caregivers: a prospective observational cohort study

**DOI:** 10.1186/s12904-016-0082-y

**Published:** 2016-01-15

**Authors:** Katja Krug, Antje Miksch, Frank Peters-Klimm, Peter Engeser, Joachim Szecsenyi

**Affiliations:** Department of General Practice and Health Services Research, University Hospital Heidelberg, Vossstr. 2, Geb. 37, 69115 Heidelberg, Germany

**Keywords:** Quality of life, Palliative care, Primary health care, Family caregiver, Observational study

## Abstract

**Background:**

Family caregivers play a key role in palliative care at home, and understanding the interdependencies in the constellation of patient, family caregivers and service providers is important. As few longitudinal studies have examined the influence of patient quality of life (QoL) in palliative care on burden of family caregivers, the aim of this study was to identify correlations between changing patient QoL and changing burden of family caregivers that need consideration in patient management.

**Methods:**

Palliative patients with cancer in primary care evaluated their QoL (Quality of Life Questionnaire Core 15 Palliative Care, QLQ-C15-PAL). They were assessed monthly for an interval of 6 months or until death of the patient. Family caregivers reported the burden they perceived while supporting the patient (Short form of the Burden Scale for Family Caregivers, BSFC). Longitudinal data were analysed for all patients with at least 3 available assessments, considering the most recent data for participants with more than 3 assessments. Changes in patient QoL were analysed using the Friedman test. In a stepwise regression analysis, influences of change in patient QoL on changing caregiver burden were investigated.

**Results:**

One hundred patients (63 men, 37 women; average age: 68 years) were enrolled in the study. The most common primary diagnoses were colon, lung or breast cancer. In 58 cases, assessments were available from both patients and caregivers. Patients reported overall quality of life increasing towards end of life, although reporting that physical functioning deteriorated. Symptoms of pain and fatigue bothered patients most. Caregiver burden was moderate and on average did not change over time. In a stepwise regression model, the difference in emotional functioning and the difference in dyspnoea showed an influence on the development of caregiver burden (explained variance of 19.3 %).

**Conclusions:**

Patients’ dyspnoea, feelings of depression and anxiety impacted on the perceived burden of family caregivers, but are manageable symptoms. Our results corroborate the need of regular assessment of patients’ needs taking into account caregiver burden. In this way, general practice teams can intervene early and may more likely meet patients’ needs in the end of life care process.

**Trial registration:**

Current Controlled Trials ISRCTN78021852, assigned on 04/04/2007

## Background

Palliative care, as defined by the World Health Organization, not only aims to maintain patients’ quality of life, but also to include the support of family caregivers [1].

Most people at the end of life wish to be cared for at home [2]. Family caregivers play an important role in the realisation of an effective home care. Nevertheless, family caregivers are confronted with various challenges, not only meeting the care needs of their relative, but also dealing with the demands on their own health, on the family and perhaps also their job situation [3]. Family caregivers support patients by giving them practical help, providing personal care, supporting them psychologically, and often taking care of medication administration [4, 5]. These caring tasks in addition to having a relative at the end of life, influence caregivers physically, emotionally, and socially. Emotionally, they have to deal with, for example, fear or loss [6]. Due to care giving, they frequently lack sleep and feel tired and exhausted [7], and may suffer from anxiety and depression [8]. Family caregivers, especially in midlife, report lower health-related quality of life than the average population [9]. Trustful and reliable relationships within their familiar and social systems are important resources for patients in palliative situations [10]. If these sources of support are exhausted palliative care at home is only possible to a limited extent.

As the WHO definition of palliative care already suggests, patient quality of life and burden of family caregivers should be considered together. Large studies and reviews are available looking at the relationship between patient QoL and caregiver burden at one time-point (i.e. a study with cancer patients [11] and a review on patients with motor neurone disease [12]), but also between unmet needs of patients and caregiver stress burden [13]. The longitudinal relationship between QoL and caregiver burden over time was described by Murray et al. on the basis of observational qualitative data [14]. To our knowledge, there are no studies trying quantitatively to show the dependence of caregiver burden on changes in patient’s quality of life in a larger sample over time.

To close this gap, this study therefore quantitatively analyses the correlation between quality of life in palliative care patients and the burden perceived by their family caregivers over a 6-month time period. The study was part of a larger project which primarily evaluated palliative CME courses for general practitioners (GPs) [15].

## Methods

In this prospective observational cohort study, palliative patients cared for at home at the end of life and their family caregivers were asked to fill in short questionnaires at monthly intervals for a period of six months or until death of the patient (if the patients died within the 6-month observation period). Patients judged their quality of life on the Quality of Life Questionnaire Core 15 Palliative (QLQ-C15-PAL) [16] of the European Organization for Research and Treatment of Cancer (EORTC). Family caregivers reported their perceived burden on the short form of the Burden Scale for Family Caregivers (BSFC) [17]. The study was conducted in compliance with the Helsinki Declaration. The study protocol was approved by the ethics committee of the Medical Faculty Heidelberg (S-043/2007). The present study is a secondary analysis of a trial that was registered (ISRCTN78021852, assigned on 04/04/2007) and its study protocol was published [15].

### Participants

Patients were eligible for inclusion in the study if they fulfilled the following criteria: being in a palliative situation with a diagnosis of advanced cancer, where the GP would not be surprised if they died within six months, and having no other disease with a lower life expectancy, and being in outpatient care by a GP who participated in the study as well. Patients and family caregivers had to be adult (at least 18 years of age) and needed a sufficient command of German to understand the study information and the questionnaires. Patients, family caregivers, and GPs had to give their informed and written consent to participate.

### Data collection

Participating GPs informed eligible patients and family caregivers from their practice about the study and asked for the patients’ and family caregivers’ consent to participate. Data collection took place between September 2007 and June 2009. After inclusion in the study, participants monthly received questionnaires containing the QLQ-C15-PAL [16] and the BSFC [17] from the GPs. This was for a follow-up period of 6 months at most. Questionnaires were sent back to the study centre in postage-paid return envelope immediately after they were filled out. For study purposes, i.e. follow-up and matching patients with their family caregivers, patients were given a pseudonym number printed on the questionnaires to ensure confidentiality. The study centre was not able to identify patients or family caregivers personally; GPs were not informed of participants’ individual answers. GPs provided data on patients’ diagnoses and performance status (Eastern Cooperative Oncology Group (ECOG)) [18].

The QLQ-C15-PAL was developed as a core instrument to measure quality of life especially in cancer patients in palliative care. It consists of 15 questions, which are transformed into 2 function scales (‘Physical Functioning’, ‘Emotional Functioning’), 7 symptom scales (‘Fatigue’, ‘Nausea/Vomiting’, ‘Pain’, ‘Dyspnoea’, ‘Insomnia’, ‘Appetite loss’, ‘Constipation’) and an ‘Overall quality of life’ scale. Patients should answer the questions according to their experiences during the prior week. Fourteen questions are answered on a 4-point Likert scale with 1 ‘Not at all’, 2 ‘A little’, 3 ‘Quite a bit’, and 4 ‘Very much’, the question to overall QoL allows answers between 1 ‘Very poor’ and 7 ‘Excellent’. The QoL, function, and symptom scales take values between 0 and 100 with higher values indicating a higher QoL, higher functioning, and higher symptom burden, respectively.

The short version of the BSFC is a 10-item questionnaire developed out of the original 28-item version for usage in general practice. Family caregivers assessed statements to their perceived burden (i.e. “I often feel physically exhausted”) on a 4-point Likert scale with 0 ‘no, definitely not’, 1 ‘no, not really’, 2 ‘yes, generally’, and 3 ‘yes, definitely’. A sum score could be calculated with scores up to 9 points indicating no to little burden, scores between 10 and 20 indicating moderate burden, and scores between 21 and 30 indicating severe to very severe burden.

### Statistical analyses

Data from family caregivers on BSFC were described both as means (M) with standard deviation (SD) and as frequencies as classified in no/little, moderate, and (very) severe burden. Nonparametric data from patients on QLQ-C15-PAL are presented as median (Md) with interquartile range (IQR). In this secondary analysis, longitudinal data were analysed for all patients with at least 3 available assessments, considering the most recent data for patients with more than 3 assessments (t3: last assessment, t2: second to last assessment, t1: third to last assessment). The Friedman test was used to detect differences over time in patient quality of life data. A clinically significant improvement or deterioration was marked by a mean difference of at least 10 points between assessments [19].

In order to assess a potential influence on the development of caregiver burden (dependent variable) related to changes of QLC-C15-PAL values for the last 3 assessment results (independent variables), we performed a multivariable linear regression. To reduce the number of independent variables, single regression analyses were performed first with each potential independent variable. Variables showing an influence (*p* < .10) were included in the multivariable linear regression. The antecedents for a multivariable linear regression were checked.

For all tests, *p* < .05 was considered to be statistically significant. All statistical analyses were conducted using SPSS 20 (IBM SPSS Statistics).

## Results

### Sample characteristics

In June 2007, a random sample of 696 GPs in south-western Germany (Federal State of Baden-Wuerttemberg) was initially invited to participate in the study. Ninety GPs consented to participate of which 47 GPs cared for eligible patients within the observation period. Eligible palliative patients of two GPs did not consent to participate in the study. The patient sample consisted of 37 women and 63 men with a mean age of 68 years. They were mainly diagnosed with colon, lung or breast cancer and had received their primary diagnosis a median of 14 months before study inclusion.

**Fig. 1:**
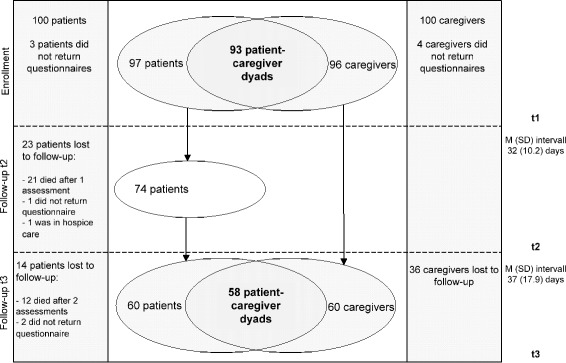
Flowchart of patients and caregivers

During the 6-month observation period, 55 patients died, 3 patients went into a hospice and were no longer cared for by their GP, and 42 patients survived the observation period. Mean time intervals between t3-t2, t2-t1 and t3-t1 assessments were 37 (SD = 17.9, range 13–107) days, 32 (SD = 10.2, range 4–79) days, and 69 (SD = 21.6, range 38–135) days, respectively. In 58 cases, t3 and t1 assessments were available from both patients and caregivers (Fig. 1). These 58 cases for the present analysis did not differ from the excluded patients, except for having had a higher performance status at study inclusion and suffering less from fatigue and appetite loss at the last available assessment. Excluded patients died earlier (before completing 3 assessments). Socio-demographic characteristics of included patients and caregivers are provided in Table 1.

**Table 1:** Characteristics of patients and caregivers in sample

		Patients	Caregivers
Women (%)		21 (36,2)	45 (77,6)
Men (%)		37 (63,8)	13 (22,4)
Age; mean yrs (SD)		70,0 (12,0)	57,1 (15,3)
Main cancer diagnosis (ICD-10) (%)	C18 (colon)	8 (13,8)	
	C34 (lung)	8 (13,8)	
	C50 (breast)	8 (13,8)	
	C61 (prostate)	5 (8,6)	
	C16 (stomach)	4 (6,9)	
	other	25 (42,1)	
Time since primary diagnosis; median months (interquartile range)		16 (3-57)	
Missing (%)		7 (12,1)	
ECOG Performance status at study inclusion (%)			
Fully active, able to carry on all pre-disease performance without restriction (grade 0)		10 (17,2)	
Restricted in physically strenuous activity but ambulatory and able to carry out work of a light or sedentary nature (grade 1)		15 (25,9)	
Ambulatory and capable of all selfcare but unable to carry out any work activities, up and about more than 50 % of waking hours (grade 2)		18 (31,0)	
Capable of only limited selfcare, confined to bed or chair more than 50 % of waking hours (grade 3)		15 (25,9)	
Completely disabled, cannot carry on any selfcare, totally confined to bed or chair (grade 4)		0 (0,0)	
ECOG Performance status at last assessment (%)			
Fully active, able to carry on all pre-disease performance without restriction (grade 0)		9 (15,5)	
Restricted in physically strenuous activity but ambulatory and able to carry out work of a light or sedentary nature (grade 1)		10 (17,2)	
Ambulatory and capable of all selfcare but unable to carry out any work activities, up and about more than 50 % of waking hours (grade 2)		16 (27,6)	
Capable of only limited selfcare, confined to bed or chair more than 50 % of waking hours (grade 3)		11 (19,0)	
Completely disabled, cannot carry on any selfcare, totally confined to bed or chair (grade 4)		11 (19,0)	
Missing		1 (1,7)	

### Caregiver burden and patient quality of life

Patients reported low overall quality of life and low physical functioning. Physical functioning deteriorated towards the end of life (*p* < 0.01) (see Table 2). However, overall quality of life increased towards end of life, but this did not reach statistical significance (*p* = 0.07) (see Table 2). Pain and fatigue bothered patients the most; nausea and vomiting were considered the least problematic. On average, all symptoms except fatigue (mean difference between assessments > 10 points) remained stable over time (Table 2). Within the patient group, large differences were reported with extreme variation of 100 points in both directions (i.e. appetite loss between t3 and t1, constipation between t2 and t1 and between t3 and t2). Least variation was observed in overall quality of life between t3 and t2 with differences ranging between -33 and 50.

**Table 2:** Patient quality of life (QLQ-C15-PAL) at three assessments (Md with IQR)

QLQ-C15-PAL dimension	Number	t1	t2	t3	p*
Overall quality of life	46	33.3 (33.3-66.7)	33.3 (16.7-66.7)	50.0 (16.7-50.0)	.07
Physical functioning	53	33.3 (13.3-73.3)	33.3 (6.7-60.0)	20.0 (0.0-73.3)	<.01
Emotional functioning	56	50.0 (41.7-66.7)	50.0 (22.9-66.7)	41.7 (4.2-66.7)	.47
Dyspnoea	54	33.3 (0.0-66.7)	33.3 (0.0-66.7)	33.3 (0.0-66.7)	.35
Pain	57	66.7 (16.7-66.7)	50.0 (16.7-66.7)	66.7 (25.0-83.3)	.23
Insomnia	57	33.3 (0.0-66.7)	33.3 (0.0-66.7)	33.3 (0.0-66.7)	.35
Fatigue	56	66.7 (36.1-97.2)	66.7 (44.4-100)	77.8 (44.4-100)	.18
Appetite loss	57	33.3 (0.0-66.7)	33.3 (0.0-66.7)	33.3 (0.0-100)	.13
Nausea/vomiting	56	16.7 (0.0-50.0)	16.7 (0.0-50.0)	16.7 (0.0-50.0)	.56
Constipation	56	33.3 (0.0-58.3)	33.3 (0.0-33.3)	33.3 (0.0-66.7)	.11

*Md* median, *IQR* interquartile range; *Friedman test

Caregiver burden at both t3 and t1 was moderate with mean sum scores of 10.3 (SD = 8.4, *n* = 55) and 11.4 (SD = 7.9, *n* = 54) respectively. Mean change between t3 and t1 for *n* = 52 caregivers for whom a sum score could be calculated was -0.9 (SD = 5.2) with differences ranging between -12 and 13 points. The mean difference was neither statistically significant nor clinically relevant. The joint distribution of the classified burden (no/little, moderate, (very) severe) is shown in Table 3.

**Table 3:** Joint distribution of the caregiver burden (BSFC) at t1 and t3 (frequencies)

		t3			
		No/little burden	Moderate burden	(Very) severe burden	
t1	No/little burden	22	3	0	25 (48.1 %)
	Moderate burden	7	8	3	18 (34.6 %)
	(Very) severe burden	0	4	5	9 (17.3 %)
		29 (55.8 %)	15 (28.8 %)	8 (15.4 %)	52 (100 %)

**Fig. 2:**
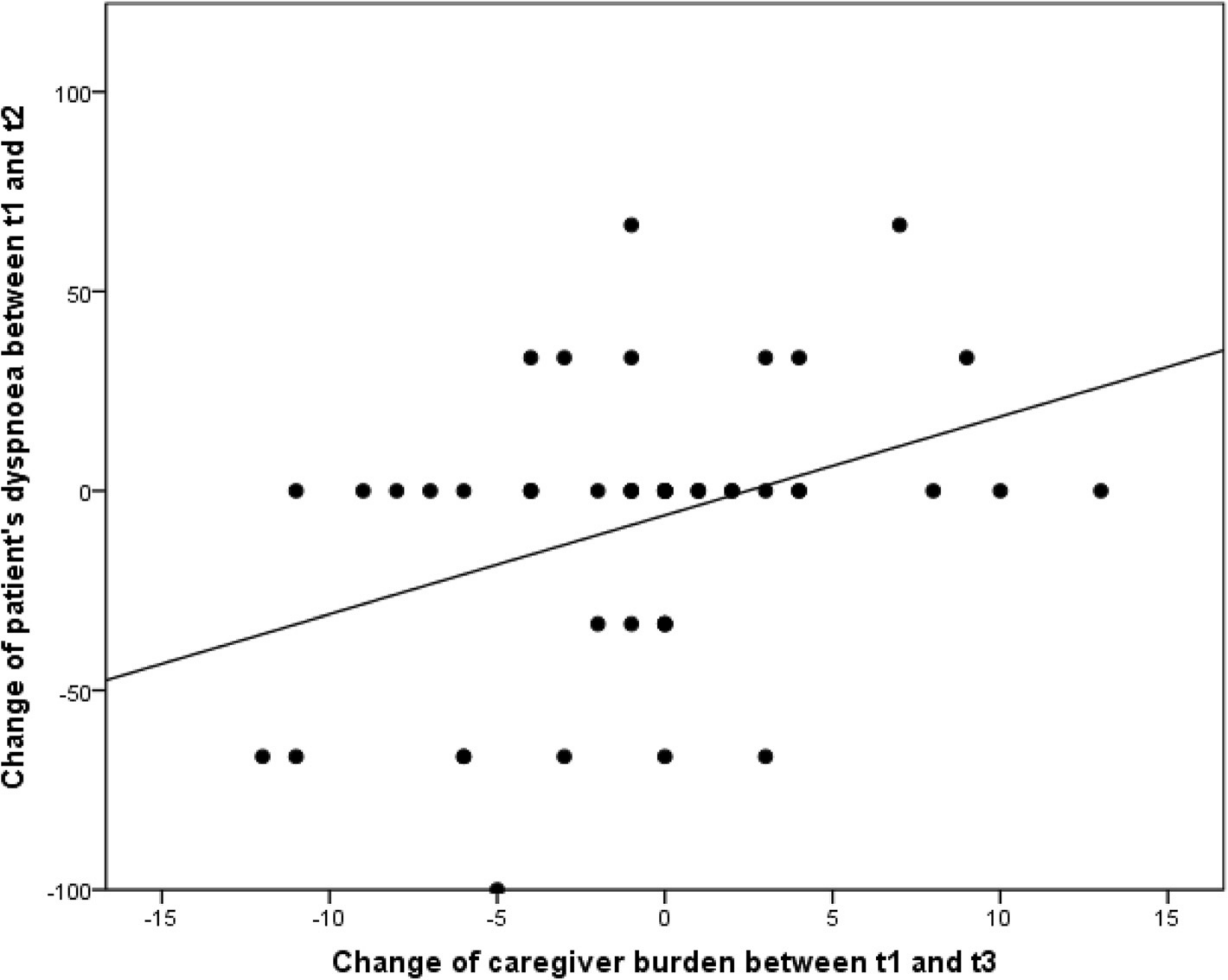
Change of caregiver burden depending on change of patient’s dyspnoea. Positive changes in caregiver burden and dyspnoea imply an increase in caregiver burden and a higher severity of dyspnoea

**Fig. 3:**
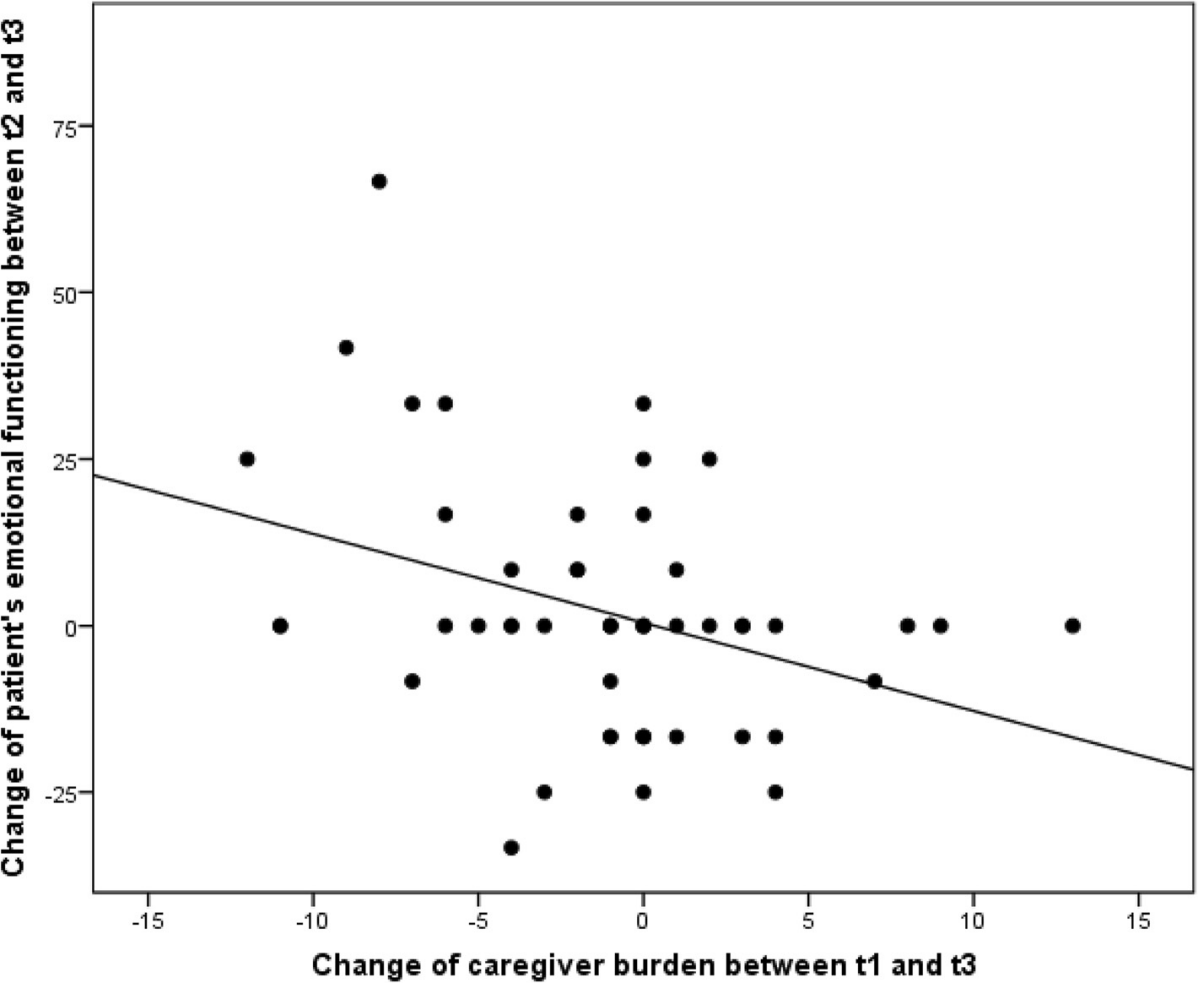
Change of caregiver burden depending on change of patient’s emotional functioning. Positive changes in caregiver burden and emotional functioning imply an increase in caregiver burden and an increase in emotional functioning

Single regression analyses showed influences on the development of caregiver burden between t3 and t1 of the following difference variables: dyspnoea between t2 and t1, dyspnoea between t3 and t1, insomnia between t2 and t1, and emotional functioning between t3 and t2. In a subsequent stepwise regression model with all four variables, the difference in emotional functioning between t3 and t2 and the difference in dyspnoea between t2 and t1 remained in the model and explained a variance of 19.3 %. Multicollinearity analysis showed variance inflation factors of less than 1.4, therefore multicollinearity did not pose a problem [20]. Caregiver burden thus increased with deteriorating emotional functioning and increasing dyspnoea of the patients (Table 4, Figs. 2 and 3).

**Table 4:** Influences on the development of caregiver burden (multivariable stepwise regression analysis): final model

Independent variables	Regression coefficient	Standard error	Confidence interval (95 %)	Regression coefficient (standardized)	*p*-value
Emotional functioning (t3-t2)	−0.08	0.04	[-0.15, -0.01]	-.31	.02
Dyspnoea (t2-t1)	0.05	0.02	[0.01, 0.09]	.30	.03
Constant	−0.44	0.67	[-2.07, 0.69]		.52

## Discussion

In a sample of palliative patients cared for at home at the end of life, the two factors of decreasing emotional functioning and increasing dyspnoea had the highest impact on increasing burden on family caregivers. For example, if patients feel increasingly tense and depressed towards the end of life, a measurable association with the burden of family caregivers was shown. Additionally, patient reported increasing dyspnoea was shown to be independently associated with increasing caregiver burden.

Other studies have reported a relation between patient and caregiver emotional status, not only for patients with cancer. Patient and caregiver anxiety and depression develop in concordance [8] and were found to be higher in family caregivers than in a non-caregiving population [21]. Patients with depression highly impact on the caregiver burden [22], while burden is also discussed to influence caregivers’ depression [23]. Our findings provide further evidence related to this issue.

Although patients in our study suffered from fatigue and pain as the most highly reported symptoms, these complaints did not influence the wellbeing of the relatives. Therefore, it remains unclear, why many people express concern about the management of pain for patients at the end of life. In addition, even though insomnia - a very disturbing symptom for somebody caring for a patient at the end of life - shows influence on the caregivers’ wellbeing, it was not responsible for the development of a higher burden score in family caregivers in our study. Often caregivers state that they worry about loss of appetite of the patients, as well as about growing nausea and vomiting and the need for prompt and sufficient help. In addition, they link appetite with the wellbeing of the patient. However, our results indicated that patient loss of appetite had no influence at all on the development of caregivers’ sense of burden. Increasing dyspnoea seemed to have a higher impact on perceived caregiver burden, although another cross-sectional study found no relationship between breathlessness and caregiver burden [24]. Since that study [24] included patients with lung cancer and heart failure, caregivers might have been more prepared for occurring breathlessness than the caregivers in our study including patients with various cancer diseases. For caregivers, breathlessness is associated with suffocation and impending death [25].

Grunfeld et al. [23] describe the relation between increasing caregiver burden and decreasing patient functional status. They evaluated first and last assessments during the palliative period with a wide time span interval between assessments. In our study, we consciously decided to analyse 3 assessments at monthly intervals within a 6-month observation period, thereby evaluating correlations over a shorter time span, which we considered appropriate in terms of the palliative care context.

Unexpectedly, there was no association between the decrease in overall patient quality of life and the increase in caregiver burden in our study. Another longitudinal study also reported not finding a relation between patients’ quality of life and caregiver well-being, which was rather influenced by caregiver functioning [26]. We did not assess caregiver functioning, so this relationship remains unclear in our study. The low association between increase in caregiver burden and the development of other patient variables, i.e. physical functioning and pain, might be due to coping strategies of family caregivers, so that they subjectively do not perceive themselves as burdened.

Based on these results, it can be recommended that general practice teams involved in the home care of palliative patients assess caregiver burden at regular intervals. This could improve support of caregivers of patients where a change in emotional function and dyspnoea is identified, since they are associated with each other. Symptom management through early interventions as well as providing information and support for both caregivers and patients is recommended. To effectively reduce caregiver burden, means not only interventions solely for caregivers, but also that interventions within the dyad are needed, managing patient symptoms as well. Early identification of patients moving into end-of-life status is of paramount importance to prevent strain on family carers and to enable support by health care professionals in this palliative constellation [27]. A regular assessment of both patient symptoms, especially dyspnoea and emotional functioning, and caregiver burden is our recommended approach to appropriately support family caregivers and to ensure good quality end-of-life care for cancer patients [10].

### Limitations of the study

Due to the study population (patients at the end of life cared for in general practices), data from only a small sample of both patients and caregivers were available for longitudinal analysis. Other explaining variables (e.g. symptoms of patients or caregiver characteristics) could potentially have been missed. Additionally, even though they were transformed to 0–100 scales, the QLQ-C15-PAL scales are in Likert response formats and therefore, as non-continuous variables, their suitability in regression analysis is limited [28].

Due to the nature of this study, the causal pathway of the observed associations cannot be determined. However, the palliative constellation with the patient being terminally ill makes it probable that the burden of the caregiver is caused by the patient’s burden, and not vice versa. Moreover, a certain selection bias cannot be excluded. In the main study, (to which the data for this secondary study is related) GPs selected patients fulfilling the eligibility criteria. It is possible that GPs potentially excluded difficult patient-caregiver dyads. Also, only patients with cancer as a primary underlying disease were included. Therefore, results cannot be generalized to other palliative care populations cared for in the home. Patients included did show substantial suffering from their disease.

## Conclusions

Our results confirm the professional requirement for general practitioners to regularly assess both patients’ needs and burden of caregivers to enable early interventions. In palliative situations, enabling patients to be cared for at home at the end of life means concerns, needs and burden of family caregivers also have to be considered as they are inter-related to each other. Caregiver burden may be eased if patients’ symptoms, especially dyspnoea, and feelings of depression and anxiety are addressed.

## Abbreviations

BSFC: Burden scale for family caregivers
CME: continuing medical education
ECOG: Eastern cooperative oncology group
EORTC: European Organisation for the Research and Treatment of Cancer
GPs: general practitioners
IQR: interquartile range
M: mean
Md: Median
QLQ-C15-PAL: quality of life questionnaire core 15 palliative
QoL: quality of life
SD: standard deviation
